# Antibody or Antibody Fragments: Implications for Molecular Imaging and Targeted Therapy of Solid Tumors

**DOI:** 10.3389/fimmu.2017.01287

**Published:** 2017-10-12

**Authors:** Katerina T. Xenaki, Sabrina Oliveira, Paul M. P. van Bergen en Henegouwen

**Affiliations:** ^1^Division of Cell Biology, Science Faculty, Department of Biology, Utrecht University, Utrecht, Netherlands; ^2^Pharmaceutics, Department of Pharmaceutical Sciences, Utrecht Institute of Pharmaceutical Sciences, Utrecht University, Utrecht, Netherlands

**Keywords:** molecular imaging, cancer therapy, antibody, antibody fragments, single-domain antibodies, nanobody

## Abstract

The use of antibody-based therapeutics has proven very promising for clinical applications in cancer patients, with multiple examples of antibodies and antibody–drug conjugates successfully applied for the treatment of solid tumors and lymphomas. Given reported recurrence rates, improvements are clearly still necessary. A major factor limiting the efficacy of antibody-targeted cancer therapies may be the incomplete penetration of the antibody or antibody–drug conjugate into the tumor. Incomplete tumor penetration also affects the outcome of molecular imaging, when using such targeting agents. From the injection site until they arrive inside the tumor, targeting molecules are faced with several barriers that impact intratumoral distribution. The primary means of antibody transport inside tumors is based on diffusion. The diffusive penetration inside the tumor is influenced by both antibody properties, such as size and binding affinity, as well as tumor properties, such as microenvironment, vascularization, and targeted antigen availability. Engineering smaller antibody fragments has shown to improve the rate of tumor uptake and intratumoral distribution. However, it is often accompanied by more rapid clearance from the body and in several cases also by inherent destabilization and reduction of the binding affinity of the antibody. In this perspective, we discuss different cancer targeting approaches based on antibodies or their fragments. We carefully consider how their size and binding properties influence their intratumoral uptake and distribution, and how this may affect cancer imaging and therapy of solid tumors.

## Introduction

Selectivity for tumor over healthy tissue is of utmost importance when it comes to successful diagnosis and treatment of cancer. Over the last several decades, great progress has been made in the development of novel targeting molecules. Traditionally, antibodies directed against antigens overexpressed in tumors are the most commonly used targeting molecules. The development of monoclonal antibody (mAb) technology together with biotechnological advances in antibody engineering has established the use of mAbs in the field of cancer (1, 2). Despite their wide success, antibody-based treatment of many solid tumors remains challenging. In most of these cases, poor efficacy is linked to non-homogeneous distribution of the mAb-based agent inside the tumor. This can subsequently result in an untargeted subpopulation of cancer cells, potentially leading to tumor relapse (3, 4). Both tumor-related factors, such as tumor microenvironment and architecture, as well as antibody characteristics contribute to this heterogeneous targeting (5). The rapidly growing field of antibody engineering has exploited the naturally occurring immunoglobulins to develop different functional antibody fragments. Modifying antibody features such as molecular size, valency, binding affinity, and pharmacokinetics allows for the development of antibody fragments with tailor-made properties for a variety of clinical applications (6, 7). A number of antibody fragments have already entered clinical trials (8) with antigen-binding fragments (Fab, ~50 kDa) and single-chain variable fragments (scFv, ~28 kDa) accounting for most of them. In the last 20–25 years, there has been a growing interest in single-domain antibodies (sdAbs) or nanobodies, the smallest naturally occurring antigen-binding fragment, consisting of the variable domain of the heavy-chain antibodies found in camelids (VHH, ~15 kDa). Their high-binding affinity, ease of production, low immunogenicity, and high stability make them a very attractive alternative to employ for targeting solid tumors. Variation in molecular size and binding properties among antibody fragments is considered to possess a central role in the intratumoral distribution of targeting molecules. In the present perspective, we aim to describe potential implications of molecular size and binding properties on tumor uptake and retention of antibody-based tracers used in molecular imaging or antibody-based therapy of solid tumors.

## From Site of Injection to Tumor Site

**Figure 1:**
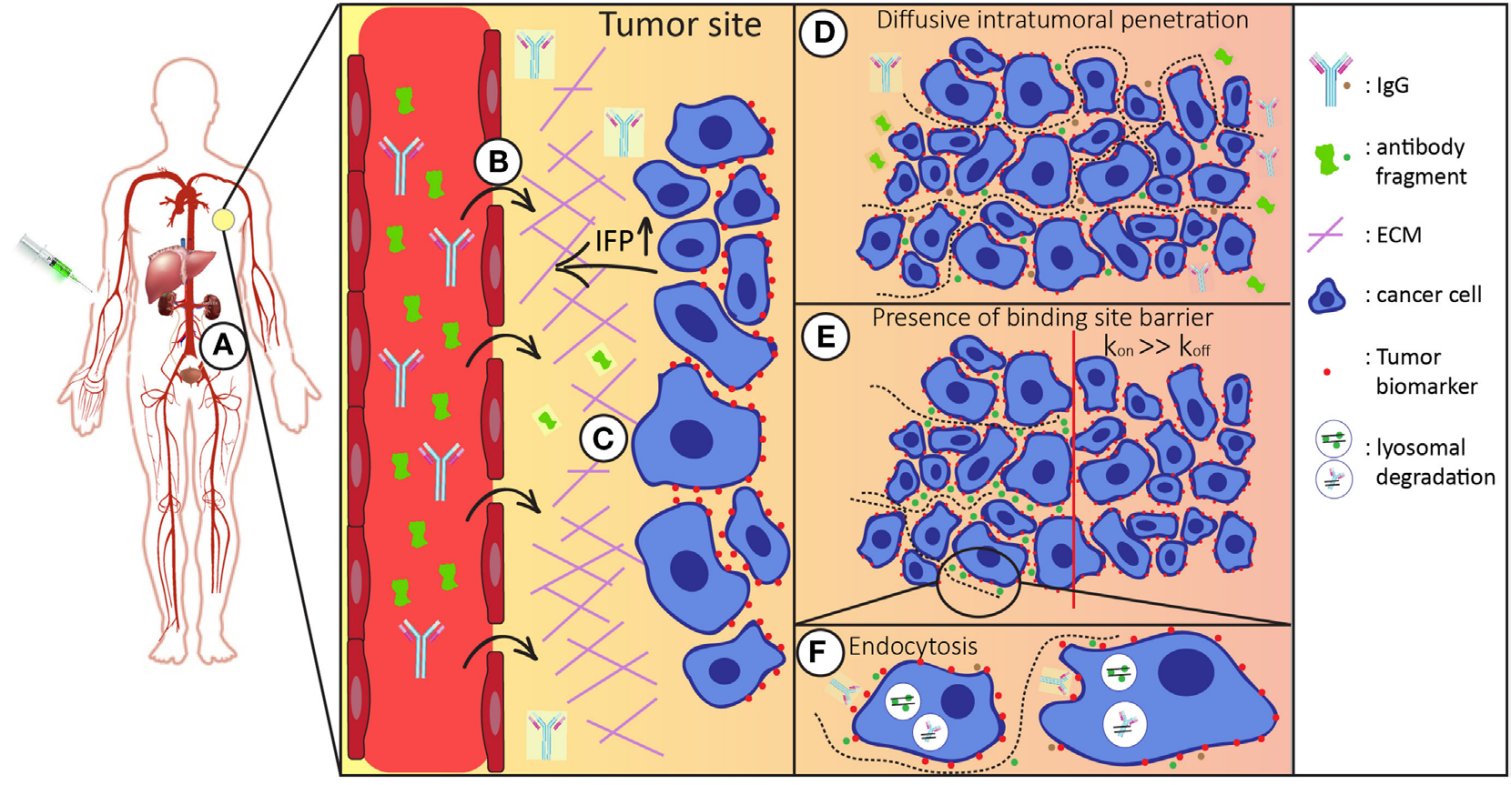
Schematic representation of the route of the antibody/antibody fragment after administration. After intravenous administration the injected antibodies/antibody fragments **(A)** enter the blood stream and circulate throughout the whole body. Both the size and binding properties of the molecule used influences tumor targeting in various ways. **(B)** It is necessary that antibodies/antibody fragments effectively extravasate into the tumor interstitium. In general, the enhanced permeability and retention (EPR) effect favors tumor accumulation of larger molecules. **(C)** In the tumor interstitium, molecules travel through tumor extracellular matrix (ECM) to reach tumor cells. Smaller molecules diffuse faster in the more densely packed ECM. **(D)** For tumor retention, the antibodies/antibody fragments should have sufficient affinity for their target molecule on the surface of the tumor cells. **(E)** Binding site barrier: molecules with high affinities have restricted penetration inside the tumor mass, which is more apparent for larger molecules. **(F)** Upon binding, antibodies are endocytosed and degraded in lysosomes. Cellular catabolism reduces the local concentration, which is the driving force of diffusive transport. Systemic clearance (*via* liver and/or kidneys) reduces the overall concentration of the administered molecules, thereby affecting intratumoral distribution.

The most common route of administration of a therapeutic antibody molecule is by intravenous injection. After entering the bloodstream, it can reach tissues throughout the body *via* blood circulation. Antibody molecules can circulate many times before they successfully extravasate at the tumor site (9). There, after they have crossed the vessel wall, they need to distribute through interstitial space and finally reach their target inside the tumor (Figure 1). Once in the tumor interstitium, molecules need to diffuse through the extracellular matrix (ECM) to reach their targets on tumor cells, where binding can take place. Their diffusion deeper inside the tumor mass largely depends on their size and antibody–antigen-binding kinetics (clearance modulus) as well as on their endocytic uptake and catabolism inside the tumor cells (Thiele modulus) (10). In addition, systemic clearance of the administered molecules lowers their concentration. Consequently, because this concentration gradient is the driving force for diffusion into the tumor, tumor accumulation is also decreased (4, 11, 12).

## Molecular Size and Vascular Permeability

After administration, the targeting antibody/antibody fragments need to cross the vascular wall of the tumor blood vessels, in order to reach the tumor cells. Properties of both the targeting macromolecule (e.g., size, shape, and charge) and the vessel wall (e.g., pore size) can influence vascular permeability [reviewed in Ref. (13)]. Mathematical modeling, using a two-pore model of the capillary wall, clearly showed that there is an inverse correlation between the size of molecules (indicated by the molecular radius) and vascular permeability (14). Experimental data measuring the vascular permeability of molecules with different molecular weights in human colon adenocarcinoma xenografts are in agreement with this prediction (15). Tumor vasculature has significant differences to that of healthy tissues. Hypervascularization and increased vascular permeability, with vessels having abnormal architecture due to the higher cell proliferation rate, as well as wider fenestrations (16) are features that promote tumor accumulation of larger macromolecules. In addition to irregular blood vasculature, there is often a lack of a proper lymphatic network inside tumors. This results in inefficient lymphatic drainage allowing for better retention of macromolecules in tumors (17, 18). This enhanced permeability and retention (EPR) effect is reported to occur in the majority of solid tumors (19). The EPR effect facilitates the accumulation of non-targeted macromolecules larger than ~40 kDa in tumors, giving an advantage to the use of larger molecules. This molecular size dependence of the EPR effect is directly linked to the systemic clearance (discussed in detail below) of the injected probes. Macromolecules larger than 40 kDa manage to circumvent renal excretion resulting in extended circulation time, increasing the chances of extravasation in the tumor interstitium (20, 21). Despite this contribution to the targeting process, the EPR effect, when present, does not guarantee sufficient and specific tumor penetration that is crucial for therapeutic applications.

Besides the improved retention at the tumor, poor lymphatic drainage also increases the interstitial fluid pressure (IFP) (17, 18). As a result, the transport of molecules into the tumor interstitium is largely dependent on diffusion rather than advective transport across the vessel wall (22). Diffusion of larger molecules is much slower, when compared to pressure-based advective transport of molecules. In cases where IFP exceeds the vascular fluid pressure, intravasation back to the blood circulation can even occur. Together, both increased IFP and abnormal tumor vasculature (leading to non-homogeneous distribution of larger molecules within the tumor) counteract the benefits of the EPR effect.

## Molecular Size and Diffusivity

The next barrier that antibodies/antibody fragments face after successful extravasation is the presence of the ECM that surrounds tumor cells. In principle, transport through ECM is based on both advective movement and diffusion, which are collectively termed convection. As a result of the elevated IFP, the pressure gradient in the tumor is considered negligible, and therefore, diffusion along a concentration gradient is the main driving force for the transport of molecules in the intercellular space. The tumor interstitium contains ECM, the composition of which differs between healthy and tumor tissues and also among tumors. The tumor ECM is often characterized by a more densely packed network of highly aligned collagen fibers, which evidently contributes to transport resistance. In both *in vivo* tumors and in multicellular spheroids, the diffusion coefficients of IgGs were lower when collagen concentration was increased (23). Next to collagen concentration, macromolecular hydrodynamic radius is inversely correlated to the diffusion coefficient. Experimental data using IgGs and dextrans of varying sizes (4–70 kDa) show decreased diffusivity when their size is larger (24, 25), which is in agreement with the Stokes–Einstein equation.

Besides the ECM composition, the arrangement of the tumor cells also contributes to geometric tortuosity, which can hinder the diffusion of macromolecules. As can be expected, diffusion rates through intercellular or interfibrillar space is directly related to the size of the diffusing molecules. Larger molecules might be restricted from diffusing within narrower tortuous intercellular paths in the same way that they are through compact collagen bundles (26, 27). Inhibition of the adherens junction protein E-cadherin (and therefore disruption of cell–cell junctions) resulted in improved penetration and cytotoxicity of an immunotoxin used in a 3D cell culture system of tumor cells (28). This is in agreement with the fact that cell density influences the distribution of anticancer agents. It has been shown that in solid tumors, consisting of more loosely packed cells, penetration of anticancer agents was faster, compared to more tightly packed cells (29).

## Effect of Molecular Size and Binding Affinity on Diffusion and Tumor Retention

An essential property of targeting molecules is their specific interaction with a cell-surface molecule on tumor cells which they bind to with high affinity. However, such binding may also contribute to heterogeneity of intratumoral distribution of antibodies. The “binding site barrier” effect, as suggested by Fujimori and colleagues, proposes that higher affinity antibodies might show restricted penetration into the tumor mass, as a result of their binding to surface located receptors (30). As seen also in studies using multicellular tumor spheroids (MTS), antibodies diffuse toward the center of the MTS covering cell layer after cell layer, which can be described as a shrinking core model. At the same time, they are able to bind to the cell surface, hindering further diffusion of unbound antibodies, therefore delaying penetration into the MTS. Diffusion into the MTS only occurs if antibody concentration is not limiting and is sufficient for saturated binding (10); thus, after all antigens have an antibody bound there would still be free molecules available to diffuse toward the center. Otherwise, when the antibody concentration is not sufficient, there will be incomplete coverage of the MTS/tumor. It has been experimentally proven, both *in vivo* and *in vitro*, that improving the affinity of the targeting molecule can result in increased tumor retention. Using anti-HER2 scFvs differing only in their dissociation constants (k_off_), Adams and colleagues demonstrated that there is a certain affinity threshold that needs to be met in order to achieve sufficient tumor retention. Even though the lower affinity scFv (K_D_ of 320 nM) showed better tumor retention than the non-binding negative control, it did not manage to achieve the high tumor accumulation levels obtained with the higher affinity scFvs (K_D_ of 16 or 1 nM) (31). However, affinity enhancement is restricted to a certain range, as beyond that no further improvement in tumor retention was documented. Mathematical modeling has shown that this affinity threshold is size dependent, with smaller molecules (~10 kDa) requiring higher affinities (0.1–10 nM) in order to reach maximum tumor uptake (14). High-affinity scFvs were at the same time shown to have less homogeneous distribution compared to ones with lower affinity (32). Similar results were obtained with scFvs targeting carcinoembryonic antigen in MTS studies, with lower affinity scFvs showing better homogeneity in their distribution (10), which is in agreement with computational modeling of antibody and antibody fragment distribution (5, 33).

One could speculate that the binding site barrier effect is more apparent when using larger molecules compared to smaller ones, provided they have similarly high affinities. In this case, there are greater chances of having local molecular crowding when using a bigger molecule consequently resulting in delay of diffusion. However, in the case of smaller fragments, one needs to make sure that they demonstrate a sufficiently high affinity (14). Otherwise, it is easier for them to diffuse back into vasculature and eventually be cleared from the system, compromising tumor retention. Other factors that may influence the binding site barrier effect are antibody catabolism by tumor cells and the dissociation kinetics (k_off_) of the antibodies. In this respect, it can be expected that a bivalent antibody, with reduced dissociation will contribute more significantly to a binding site barrier effect than the monovalent fragments.

## The Thiele Modulus and Systemic Clearance

As mentioned above, the driving force for diffusion of an antibody or antibody fragment through the vasculature and into the interstitial space of a solid tumor is the concentration gradient. This concentration can be compromised both inside the tumor (because of endocytic uptake followed by degradation within tumor cells) as well as outside the tumor (because of systemic elimination of the antibody). Antibody clearance is therefore of great importance for its distribution within the tumor, as lowering the available antibody concentration results in its decreased penetration into tumor tissue (11, 14). Intracellular degradation of the antibody largely depends on the internalization rate of the targeted tumor antigen, provided that this eventually results in the endocytosis of the targeting antibody and degradation in lysosomes. The Thiele modulus (φ^2^), describing the ratio between internalization rate and diffusion/binding rates, determines whether the administered antibody successfully targets the whole tumor. Only when diffusion is faster than clearance (φ^2^ < 1) will the moving antibody front successfully reach the core of the tumor.

Unlike endocytic catabolism, systemic clearance of the targeting molecule is dependent on its size or hydrodynamic radius. An increase in the molecular radius leads to a rapid decrease of the plasma clearance (14). In general, globular molecules smaller than ~40 kDa (hydrodynamic radii 2–6 nm) are rapidly removed from the body *via* renal filtration (34, 35). The larger IgG antibodies exhibit longer circulation times, with serum half-life of 2–3 weeks. This is due to their large hydrodynamic volume as well as to their interaction with the FcRn receptor. IgGs bound to the FcRn receptors on endothelial cells are internalized and recycled back to the cell surface (36). This prevents them from lysosomal degradation, prolonging their residence time in the circulation, with IgGs being detected to be present in the body for around 30 days after administration (37). Smaller antibody fragments exhibit shorter plasma half-lives not only because of their smaller size but also because of their inability to bind FcRn. Fab fragments, with a molecular weight of approximately 50 kDa, have been demonstrated to have a half-life of around 28 min (38); smaller scFv fragments, in the range of 30 kDa, have been shown to be cleared even faster, with plasma half-lives of only 10 min. Engineering of multivalent Fvs that results in tracers of larger size has proven to be efficient for the improvement of their half-lives (39, 40). Plasma half-life of a non-relevant radiolabeled nanobody has been calculated to be around 2 h (41), while targeting nanobodies are shown to be completely eliminated from the blood 24 h post-administration, with some levels still present mainly in the kidneys (42, 43). This short half-life of smaller antibody fragments is suggested to be beneficial, when they are used as radiolabeled tracers for imaging, to reduce the exposure of patients to ionizing radiations. Nanobodies have been shown to accumulate rapidly into tumors resulting in high tumor-to-background ratios already obtained 3–5 h postinjection.

When intended for use in therapeutic applications, half-life extension should be considered. Most of the strategies used for half-life extension of antibody fragments exploit the long half-life of serum albumin (36). Fusion to albumin targeting nanobodies (44–46) or even albumin itself (47) has proven to be successful for the half-life extension of nanobodies and scFvs. As an alternative, and with the intention to keep the size of the targeting moiety the smallest possible, fusion to albumin-binding peptides (48) or albumin-binding domains of streptococcal proteins have been used (49, 50).

## Concluding Remarks

Antibodies or larger antibody fragments show sufficient tumor uptake, regardless of their binding affinity for tumor-associated antigens, due to their longer circulation time. Their longer half-life is advantageous for therapeutic applications, as it grants them a better chance to accumulate at the tumor. At the tumor site, a longer half-life allows for a longer time for the therapeutic drug to act on the tumor cells, increasing the therapeutic index. Because of their long half-lives in combination with the EPR effect, tumor accumulation of even non-targeted macromolecular antitumor agents can be achieved. However, this comes with the cost of an uneven intratumoral distribution of such large molecules, which could potentially result in incomplete tumor cell targeting followed by tumor recurrence. Smaller antibody fragments, such as Fabs, scFvs, and nanobodies, have a more rapid tumor accumulation because of their better diffusion into the tumor as a result of their small size and different binding properties (i.e., valency), which reduce the binding site barrier effect. Similarly, non-natural binding scaffolds, such as DARpins [~14 kDa; reviewed in Ref. (51)], affibodies [~58 aa residues, 6 kDa; reviewed in Ref. (52, 53)], and non-immunoglobulin based peptides [reviewed in Ref. (54)], having even smaller sizes than nanobodies, are being successfully employed as targeting agents. As such, small antibody fragments and scaffolds, with high binding affinities, are ideal to use as imaging tracers. High tumor-to-background ratios are achieved at earlier time points and as a result of their rather quick clearance the radioactive burden of the patient in case of nuclear imaging is reduced.

When small targeting proteins are to be employed as therapeutic agents, modifications in order to improve their half-life and binding affinity are required. Smaller molecules engineered to have a longer half-life would have longer residence time in the patient and likely a more homogeneous distribution throughout the tumor. In addition, antibody fragments allow the relatively easy generation of multivalent or multispecific molecules with different binding kinetics or specificities [reviewed in Ref. (55, 56)]. This can be accomplished by genetic fusion of the respective binding domains interspaced with artificial linkers (40, 45, 57, 58). In conclusion, parameters such as affinity, valency, antigen density, antibody catabolism, and half-life extension should all be considered when choosing the appropriate antibody-based targeting agent to be used. When they have the right properties, smaller fragments can have a considerable advantage over larger ones.

## Author Contributions

KX prepared the manuscript, and SO and PH edited it.

## Conflict of Interest Statement

The authors declare that the authors KX and SO have no commercial or financial relationships that could be construed as a potential conflict of interest. PBH is shareholder of QVQ BV, Utrecht, the Netherlands.
